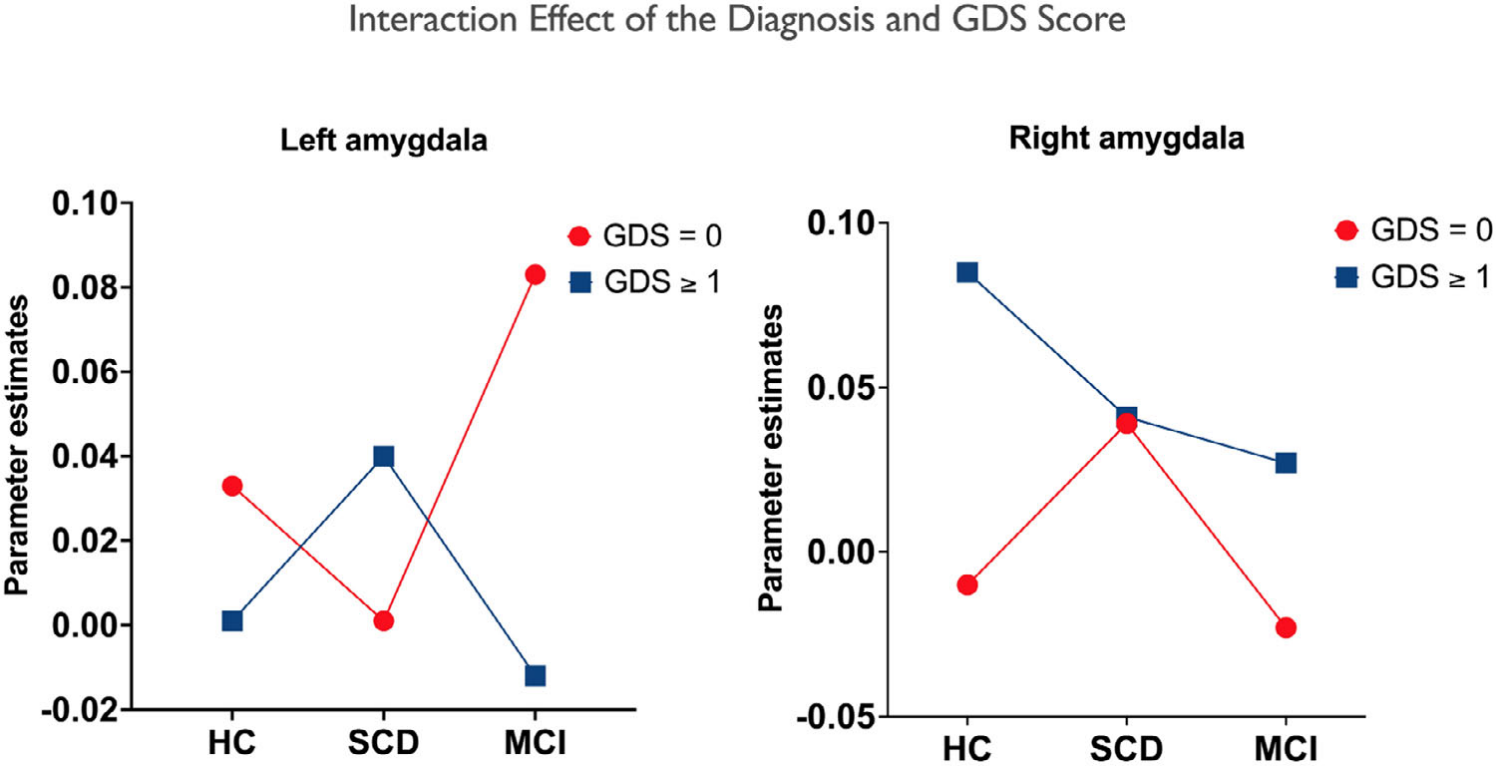# Amygdala Novelty Responses, Depressive Symptoms, and Tau Pathology in Preclinical Alzheimer's Disease: An fMRI Investigation

**DOI:** 10.1002/alz70855_107337

**Published:** 2025-12-24

**Authors:** Emrah Düzel, Ugur Cikrikcili, Björn H. Schott, Thomas Nickl Jockschat, David Berron, Joram Soch, Friedrich Krohn

**Affiliations:** ^1^ German Center for Neurodegenerative Diseases (DZNE), Magdeburg, Germany; ^2^ Institute of Cognitive Neurology and Dementia Research (IKND), Otto‐von‐Guericke University, Magdeburg, Germany; ^3^ Otto von Guericke University, Magdeburg, Sachsen Anhalt, Germany; ^4^ German Center for Neurodegenerative Diseases (DZNE), Germany, Magdeburg, Sachsen Anhalt, Germany; ^5^ German Center for Neurodegenerative Diseases (DZNE), Göttingen, Germany; ^6^ Otto von Guericke University Department of Psychiatry and Psychotherapy, Magdeburg, Sachsen Anhalt, Germany; ^7^ German Center for Neurodegenerative Diseases (DZNE), Goettingen, Germany

## Abstract

**Background:**

Late‐life depression (LLD) is a well‐established risk factor for Alzheimer's disease (AD) and has been linked to cognitive decline. The amygdala, an important region for emotional regulation and novelty detection, is one of the earliest sites of tau accumulation in AD. However, it remains unclear how depressive symptoms and tau burden interact with amygdala function in preclinical AD stages. This study examines the relationship between amygdala novelty responses, depressive symptoms.

**Method:**

A total of 190 participants (HC = 58, SCD = 88, MCI = 44) underwent functional MRI (fMRI) to assess amygdala novelty responses. Depression severity was measured using the GDS, and tau pathology was classified based on CSF phosphorylated tau (*p*‐Tau) and total tau (t‐Tau) levels. Participants were further categorized based on depressive symptoms (GDS = 0 vs. GDS ≥1) and tau status (Tau (‐) vs. Tau (+)) for statistical analyses.

**Result:**

Group comparisons revealed significantly higher GDS scores in SCD and MCI groups compared to HC (*p* < 0.05), reflecting increased depressive symptoms in individuals with cognitive complaints. However, no significant correlation was observed between amygdala novelty responses and GDS scores across diagnostic groups. Additionally, CSF tau levels (*p*‐Tau and t‐Tau) did not significantly interact with depressive symptoms in predicting amygdala responses. The lack of significant findings in amygdala response may be attributed to the relatively low overall severity of depressive symptoms in the sample, which could limit detectability of neural alterations.

**Conclusion:**

Although prior studies have indicated that late‐life depression and tau pathology are associated with the progression of Alzheimer's disease (AD), our findings suggest that subclinical depressive symptoms do not significantly impact amygdala novelty responses in preclinical AD. The observed increase in depressive symptoms in SCD and MCI groups suggests that affective changes may still be an important factor in disease progression, but their relationship with amygdala function remains unclear. Future research should explore clinically significant depression, longitudinal changes in affective symptoms, and other neurobiological mechanisms, such as amyloid burden and neuroinflammation, to better understand the early neural changes associated with AD.